# Transcriptome Profile Analysis from Different Sex Types of *Ginkgo biloba* L.

**DOI:** 10.3389/fpls.2016.00871

**Published:** 2016-06-16

**Authors:** Shuhui Du, Yalin Sang, Xiaojing Liu, Shiyan Xing, Jihong Li, Haixia Tang, Limin Sun

**Affiliations:** Key Laboratory of Sivilculture in Shandong Province, College of Forestry, Shandong Agriculture UniversityTai'an, China

**Keywords:** *Ginkgo biloba*, sex determination, RNA-sequencing, plant hormone signal and transduction, DNA methyltransferase, hydroxysteroid dehydrogenase

## Abstract

In plants, sex determination is a comprehensive process of correlated events, which involves genes that are differentially and/or specifically expressed in distinct developmental phases. Exploring gene expression profiles from different sex types will contribute to fully understanding sex determination in plants. In this study, we conducted RNA-sequencing of female and male buds (FB and MB) as well as ovulate strobilus and staminate strobilus (OS and SS) of *Ginkgo biloba* to gain insights into the genes potentially related to sex determination in this species. Approximately 60 Gb of clean reads were obtained from eight cDNA libraries. *De novo* assembly of the clean reads generated 108,307 unigenes with an average length of 796 bp. Among these unigenes, 51,953 (47.97%) had at least one significant match with a gene sequence in the public databases searched. A total of 4709 and 9802 differentially expressed genes (DEGs) were identified in MB vs. FB and SS vs. OS, respectively. Genes involved in plant hormone signal and transduction as well as those encoding DNA methyltransferase were found to be differentially expressed between different sex types. Their potential roles in sex determination of *G. biloba* were discussed. Pistil-related genes were expressed in male buds while anther-specific genes were identified in female buds, suggesting that dioecism in *G. biloba* was resulted from the selective arrest of reproductive primordia. High correlation of expression level was found between the RNA-Seq and quantitative real-time PCR results. The transcriptome resources that we generated allowed us to characterize gene expression profiles and examine differential expression profiles, which provided foundations for identifying functional genes associated with sex determination in *G. biloba*.

## Introduction

Sexual phenotypes in plants are exceedingly diverse and understanding the mechanisms underlying sex determination will require data from ecology, developmental biology, and genetics (Ming et al., 2011). Hermaphroditic or monoecious species make up 94% of all flowering plants, while dioecious species make up only 4% (Renner and Ricklefs, 1995). Dioecious species are ideal models for studying sex determination. Species such as *Silene latifolia* have been extensively investigated and several sex-linked genes have been identified and isolated (Matsunaga et al., 1996; Guttman and Charlesworth, 1998; Moore et al., 2003; Wu et al., 2010). Nevertheless, sex determination in plants is a complex process that involves various genes that are differentially and/or specifically expressed in different developmental phases, the identification and characterization of only a scant handful of involved genes will not provide a full understanding of the mechanism of sex determination (Charlesworth, 2002; Charlesworth and Mank, 2010). Previously used transcriptome techniques, such as expressed sequence tags (ESTs) and microarray analysis (Irizarry et al., 2005; Terefe and Tatlioglu, 2005; Akao et al., 2007; Andersen et al., 2008), suffered from a limited depth of coverage and sensitivity as well as background or cross-hybridization problems that restricted their applications in fully elucidating the functional complexity of plant sex determination. Furthermore, it is more difficult to identify candidate genes in non-model plant species that the total genome sequences are unknown. The introduction of high-throughput RNA-sequencing (RNA-Seq) technology for transcriptome analysis has provided a more powerful and cost-efficient approach (Wang et al., 2009, 2010). RNA-Seq has been used extensively and successfully in transcriptome analyses of willow (Liu et al., 2013), wheat (Yang et al., 2015) and cucumber (Wu et al., 2010) to greatly accelerate the understanding of the complexity of gene expression, regulation, and networks in plant sex determination.

**Figure 1:**
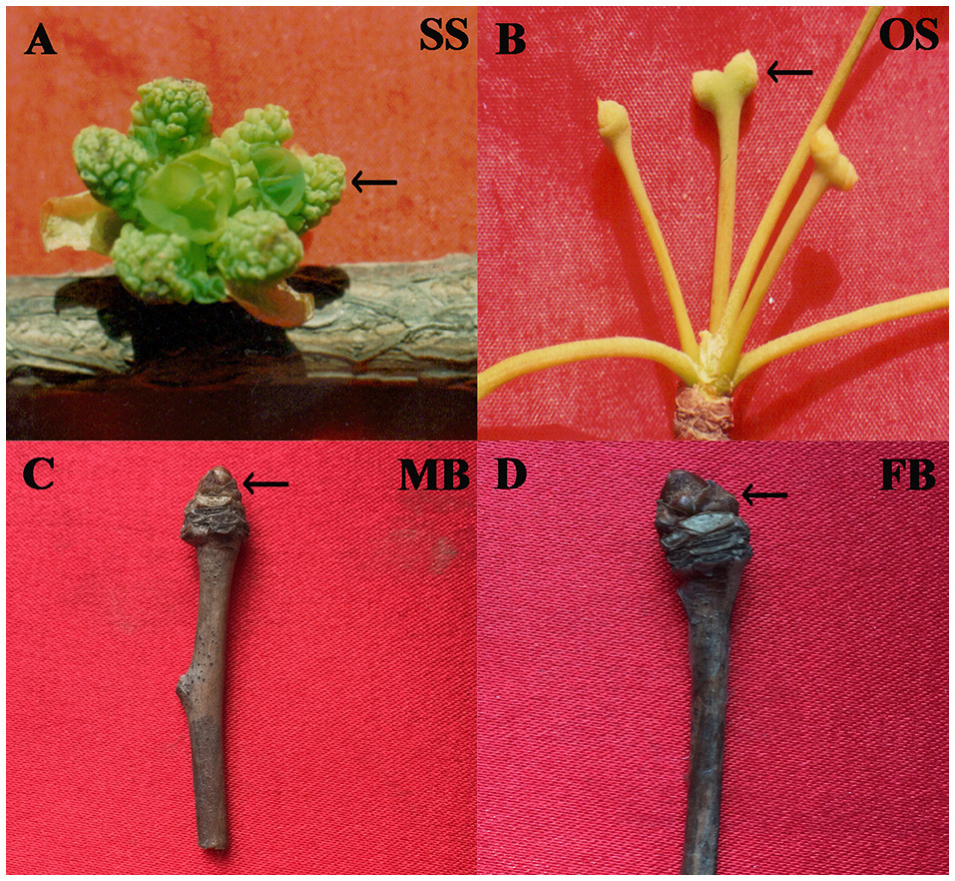
**Samples used in this study. (A)** Staminate strobilus (SS); **(B)** Ovulate strobilus (OS); **(C)** male bud (MB); **(D)** female bud (FB).

*Ginkgo biloba* L. (also referred as the “golden living fossil”) is a typical dioecious gymnosperm species with great economic and ecological values (Zhang et al., 2015). Like other gymnosperms such as species of Cycadopsida and Gnetales, the reproductive organ of male ginkgo individual is staminate strobilus (male cone) while female one bears ovulate strobilus (female cone) (Wu, 1999) (Figures 1A,B). The morphological traits, growth habits, and physiological characteristics are significantly different between mature female and male individuals, which directly influence the practical uses of this plant (Huang et al., 2013). Male individuals are mainly used in ornamental horticulture and wood industry while female ones are used for seed production. These differences are directly derived from sex determination and the following sex differentiation in *G. biloba*, however, sex determination has not yet been explored in this species. Furthermore, to our knowledge, previous and recent genomic studies on *G. biloba* mainly focused on genes involved in ginkgolides and/or bilobalide biosynthesis (Han et al., 2015; He et al., 2015), no genome-wide analyses to systematically characterize gene expression levels and to compare differentially expressed genes between male and female reproductive organs has been conducted so far. Such information is essential to understand the mechanism of sex determination in this species. Considering that sex determination involves genes expressed in different developmental stages, in the present study we used RNA-Seq to investigate and compare the transcriptomes of buds (Figures 1C,D) and strobilus of *G. biloba*. Genes related to phytohormone signal and transduction, DNA methylation were found to be differentially expressed between male and female organs. Our results provided valuable transcriptome resources for a non-model gymnosperm species and may help to reveal clues for further investigations on plant sex determination.

## Materials and methods

### Plant materials and RNA extraction

The *G. biloba* plants used in this study were planted in the Forestry Experimental Field of Shandong Agriculture University located in Taian, Shandong Province, China (35°38′−36°28′ N, 116°02′−117°59′ E). Five male trees and five female ones of 25-year-old from the same family were chosen to exclude stochastic error caused by different genetic background. For female and male buds as well as ovulate strobilus and staminate strobilus, two samples were collected from each tree on 4th and 28th March 2015, respectively, based on the development stages categorized previously (Shi et al., 1998; Zhang W. P. et al., 2001). For the buds, the bud bracts were removed immediately after sampling. All the materials were frozen in liquid nitrogen and stored at −80°C till used.

For each group of materials, five samples from different trees of the same gender were pooled to form one biological sample [two biological replicates were performed for each group as in other studies (Frey et al., 2015; Yang et al., 2015)] for total RNA extraction using TRIZOL reagent according to the manufacturer's instructions (Invitrogen, USA). The quality and quantity of the total RNA were assessed using 1% agarose gels and a NanoPhotometer® spectrophotometer (Implen, CA, USA). Subsequently, genomic DNA was digested by treatment with DNase I and mRNA was isolated using Dynabeads Oligo (dT) (Invitrogen, USA). Then, the concentration and integrity of the mRNA were quantified using a Qubit® RNA Assay Kit in Qubit 2.0 Fluorometer (Life Technologies, CA, USA) and RNA Nano 6000 Assay Kit in an Agilent Bioanalyzer 2100 system (Agilent Technologies, CA, USA).

### Libraries construction, deep sequencing, and *de novo* assembly

Eight transcriptome sequencing libraries were generated using a NEB Next® Ultra™ RNA Library Prep Kit for Illumina® (NEB, USA) following the manufacturer's recommendations. The libraries were sequenced on an Illumina HiSeq 2500 platform. To obtain high-quality clean reads for assembly, the raw reads were filtered through in-house perl scripts by removing adaptor sequences, reads containing poly-N sequences, and low quality reads. All the downstream analyses were conducted using the clean read sequences. All the clean reads were pooled and assembled using the Trinity *de novo* assembly program (Grabherr et al., 2011) with the minimum kmer_cov set to 2 as the default, and all other parameters set to their default values (He et al., 2015).

### Functional annotation

The assembled unigenes were searched against the Nr and Nt databases, and the Swiss-Prot protein and COG/KOG databases using BLAST with an cutoff *E*-value of 1e^−5^. To assign functional annotations, the unigenes were searched against Pfam using HMMER 3.0 (Finn et al., 2011) with *E*-value of 1e^−2^, the KEGG database using KAAS (KEGG Automatic Annotation Server) (Moriya et al., 2007) with *E*-value of 1e^−10^, and the GO database using Blast2GO (Gotz, 2008) with an *E*-value of 1e^−6^.

### Identification of differentially expressed genes (DEGs)

For all the comparisons, read counts were normalized by calculating the FPKM value (Trapnell et al., 2010) to obtain relative expression levels. An FPKM value >0.3 was defined as the threshold of significant gene expression. Differential expression analysis was performed using the DESeq R package 1.10.1 (Anders, 2010), which provides statistical routines for determining differential expression in digital gene expression data using a model based on the negative binomial distribution. The resulting *P*-values were adjusted using the Benjamin and Hochberg's approach for controlling the false discovery rate (Storey and Tibshirani, 2003). Unigenes with an adjusted *P* < 0.05 determined by DESeq were assigned as DEGs. GO enrichment analysis of the DEGs was performed using the GOseq R packages based on the Wallenius' non-central hypergeometric distribution (Young et al., 2010), which can adjust for gene length bias in DEGs. We also used the KOBAS software (Mao et al., 2005) to test the statistical enrichment of DEGs in the KEGG pathways.

### qRT-PCR validation

Based on the classification of genes and/or pathways revealed in this study (see below), we selected 26 genes from different categories generated by RNA-Seq for validation. We designed specific primers that corresponded to the conserved region of each cDNA in the sequenced database (Table S1). Real-time assays were performed with SYBR Green Dye (Takara, Dalian, China) using a Bio-Rad CFX96 real-time PCR platform (BioRad Laboratories, Hercules, CA, USA) with the following cycle conditions: 95°C for 5 min, followed by 45 cycles of 95°C for 10 s, 60°C for 10 s, and 72°C for 20 s. Three biological replicates were used for each gene. RNA transcript fold changes were calculated using the 2^−Δ*ΔCt*^ method (Livak and Schmittgen, 2001) with *GAPDH* as the internal control (Wang et al., 2012).

## Results

### Sequencing analysis and *de novo* assembly

Eight cDNA libraries were constructed using female and male buds (FB and MB) as well as ovulate strobilus and staminate strobilus (OS and SS) of 25 year-old *G. biloba* trees. The libraries were sequenced on an Illumina Hiseq 2500 platform. The sequences of the raw reads have been deposited in NCBI Sequence Read Archive (SRA) under accession numbers SRR2147720 (SS), SRR2147715 (OS), SRR2147717 (FB), and SRR2147721 (MB). After the raw reads were filtered, 119,494,172 (MB), 115,958,434 (FB), 121,730,470 (SS), and 116,504,860 (OS) clean reads were obtained (Table S2). All the clean reads were pooled together and assembled *de novo* using Trinity (Grabherr et al., 2011). A total of 108,307 unigenes with an average length of 796 bp and a N50 of 1648 bp were obtained, among which 23,624 unigenes (21.81%) were longer than 1000 bp. The length distribution of the unigenes is shown in Figure S1. The fasta file of the unigene set is available from the Dryad Digital Repository (http://dx.doi.org/10.5061/dryad.nb028). To evaluate the efficiency of short-read usage during the *de novo* assembly, the clean reads were mapped back onto the unigenes using RSEM (Bo and Dewey, 2011). A total of 100,682,078 (MB), 98,967,076 (FB), 104,142,618 (SS), and 99,124,608 (OS) sequences were mapped (~85%), which indicated that the assembled unigenes could be used for the subsequent analysis.

### Functional annotation of the unigenes

The unigenes were matched against sequences in the Nr, Nt, Swiss-Prot, COG/KOG, Pfam databases to assign functional annotations. A total of 6347 (5.86%) unigenes found matches in all the searched databases and 51,953 (47.97%) unigenes matched sequences in at least one of the databases; 56,354 (52.03%) unigenes did not match any of the known sequences in the public databases (Table 1 and Table S3). The number of unigenes assembled and annotated varied between the present study and other transcriptomic studies of *G. biloba* (Han et al., 2015; He et al., 2015), which may result from the novel genes specially expressed in different organs, or they may be the result of technical or biological biases (Rao et al., 2014).

**Table 1:** **Summary of functional annotation of the unigenes**.

	**Number of unigenes**	**Percentage (%)**
Annotated in NR	40494	37.38
Annotated in NT	19771	18.25
Annotated in KO	15216	14.04
Annotated in SwissProt	35521	32.79
Annotated in PFAM	34700	32.03
Annotated in GO	35190	32.49
Annotated in KOG/COG	19000	17.54
Annotated in all Databases	6347	5.86
Annotated in at least one Database	51953	47.96

**Figure 2:**
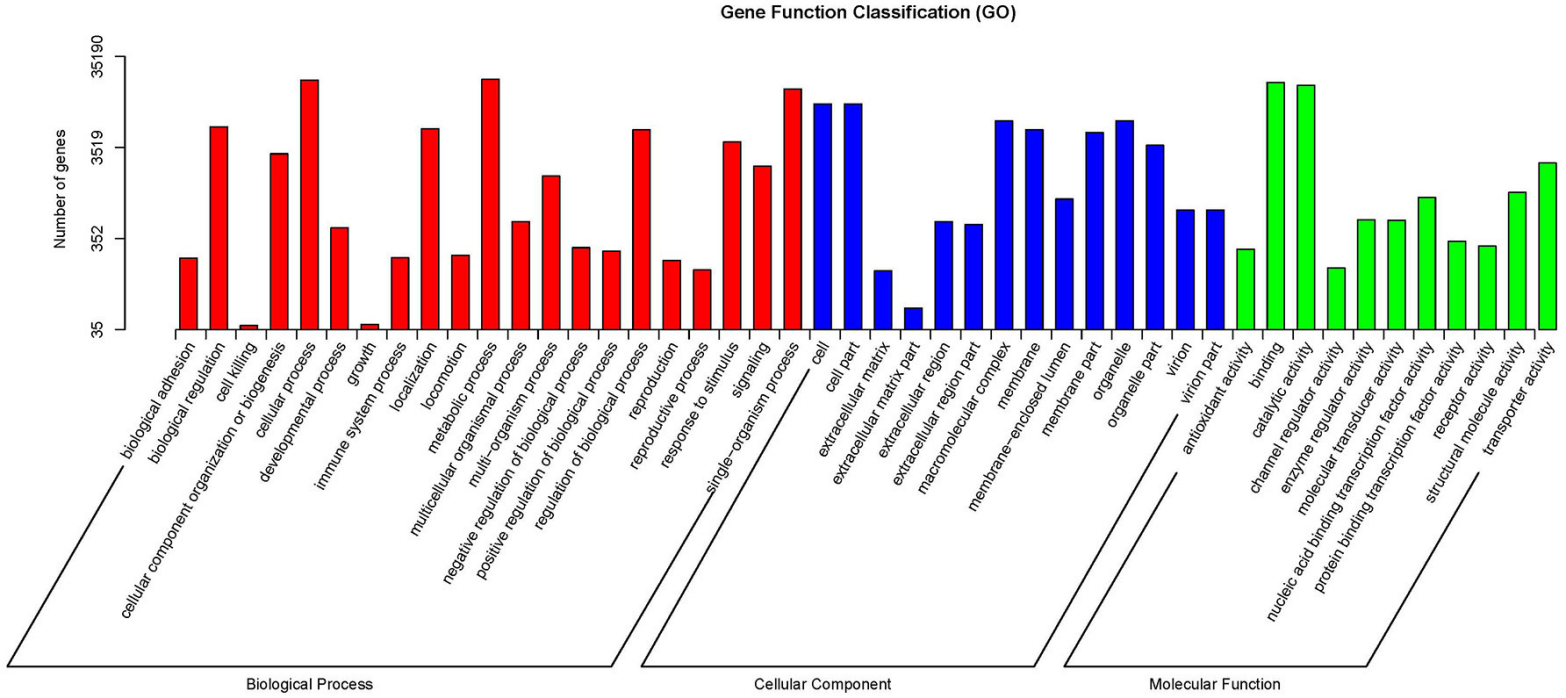
**Gene ontology (GO) functional classification of unigenes**.

**Figure 3:**
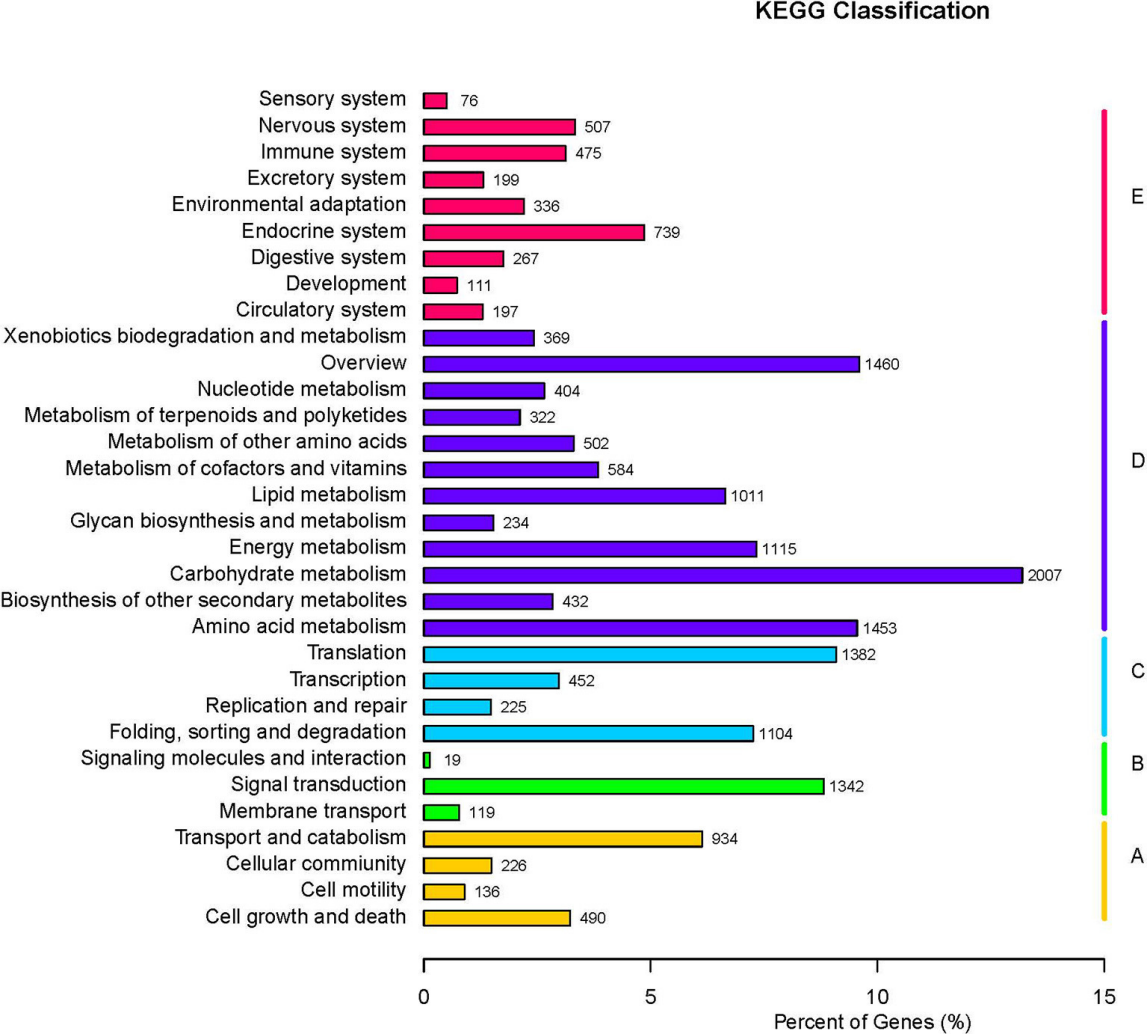
**Kyoto encyclopedia of genes and genomes (KEGG) classification of unigenes**.

The GO database was searched using Blast2GO (Conesa et al., 2005) to functionally classify the unigenes. In several cases, multiple categories were assigned to the same unigene. A total of 180,424 terms were assigned under 23 subcategories of biological process, 14 subcategories of molecular function, and 18 subcategories of cellular component (Figure 2). The KEGG pathway database was used to predict the biological functions and the interactions of the gene products. Overall, 15,216 unigenes were assigned to 32 KEGG pathways (Figure 3).

### Analysis of DEGs

**Figure 4:**
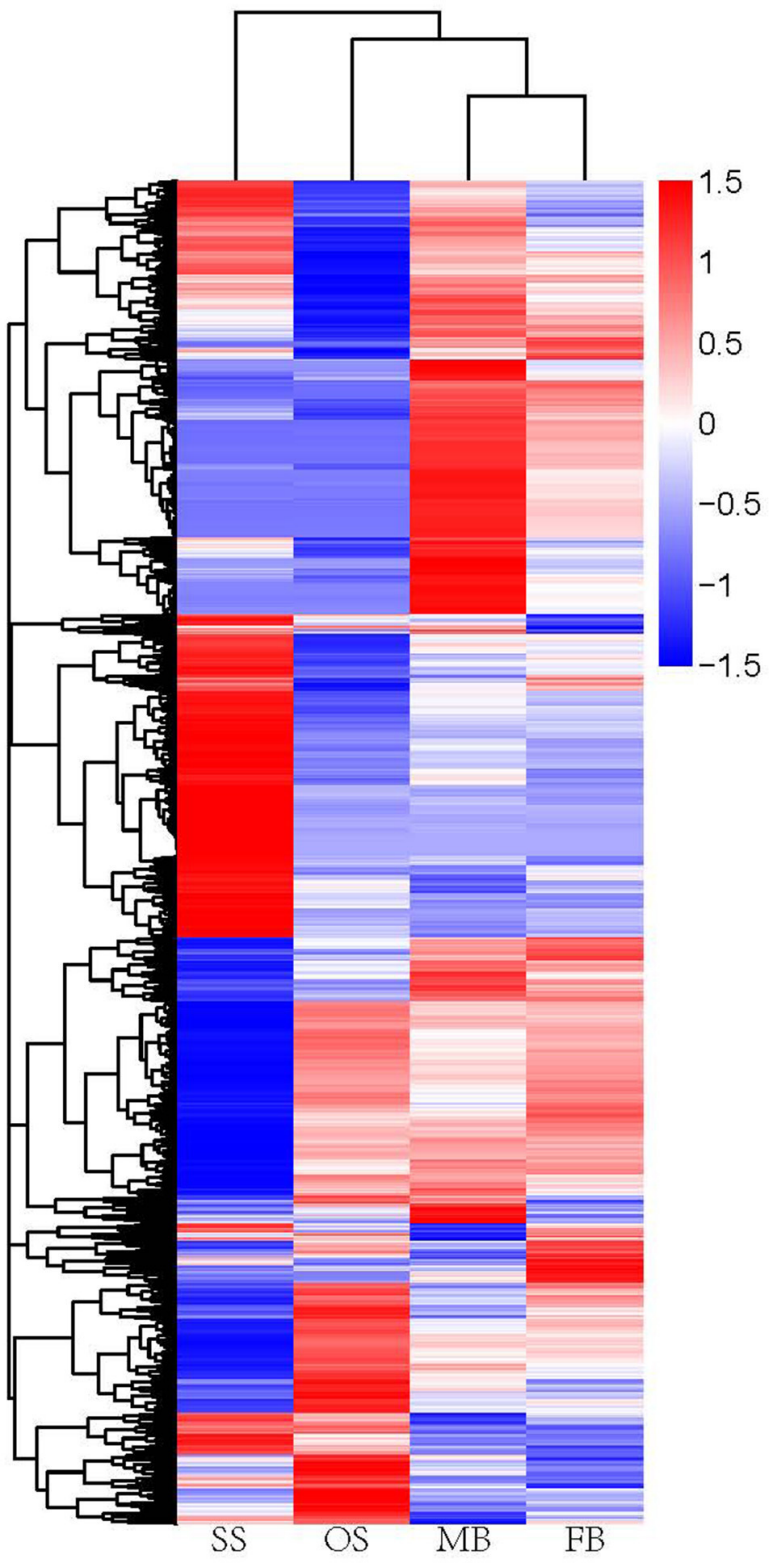
**Heatmap of hierarchical clustering of DEGs**. Each column represens a sample, each row represents a unigene. The differences in expression level were shown in distinct colors. Positive numbers indicate up-regulation DEGs and negative number indicates down-regulated DEGs. SS, staminate strobilus; OS, ovulate strobilus; MB, male bud; FB, female bud.

To identify unigenes potentially involved in sex determination of *G. biloba*, we compared the transcriptome profiles between FB and MB, and between OS and SS. The total numbers of differentially expressed genes (DEGs) in MB vs. FB and SS vs. OS were 4709 and 9802 respectively, in which 1944 unigenes were commonly expressed (Figure 4), with an adjusted *P* < 0.05 as the threshold. In MB vs. FB, 2235 genes were up-regulated and 2474 were down-regulated, while in SS vs. OS, 5849 genes were up-regulated and 3953 were down-regulated.

GO enrichment analysis of the DEGs in MB vs. FB showed that they were enriched in 36 categories with metabolic process (GO:0008152; 2154 unigenes) and cellular metabolic process (GO:0044237; 1678 unigenes) representing the most abundant categories, followed by cell part (GO:0044464; 1241 unigenes) and cellular macromolecule metabolic process (GO:0044260; 1222 unigenes) (Figure S2). Among the 9802 DEGs in SS vs. OS, 3115 were enriched in 18 categories. Cellular component biogenesis (GO:0044085; 427 unigenes) and ribonucleoprotein complex biogenesis (GO:0022613; 272 unigenes) were the most abundant categories, followed by ribosome biogenesis (GO: 0042254; 268 unigenes) and hydrolase activity, acting on glycosyl bonds (GO:0016798; 233 unigenes) (Figure S3). The most enriched KEGG pathways in SS vs. OS were ribosome (143 unigenes) and starch and sucrose metabolism (97 unigenes), followed by plant hormone signal transduction (76 unigenes) and phenylpropanoid biosynthesis (67 unigenes) (Figure S4). Among the 76 DEGs annotated to be involved in plant hormone signal and transduction (Table S4), 11 DEGs were commonly differentially expressed in MB vs. FB and SS vs. OS (Table 2, Data sheet 1), which might be involved in sex determination of *G. biloba*. In MB vs. FB, ribosome (190 unigenes) and phenylpropanoid biosynthesis (33 unigenes) were the most enriched pathways (Figure S5).

**Table 2:** **The fold changes and KO annotations of 11 commonly DEGs in MB vs. FB and SS vs. OS**.

**Gene ID**	**Log_2_FC^a^(SS vs. OS)**	**P adj^b^**	**Log_2_FC(MB vs. FB)**	**P adj**	**KO name**	**KO description**
c19444_g1	4.2839	1.73E-12	1.2588	4.77E-05	SAUR	SAUR family protein
c27303_g1	−1.1861	0.010399	−.61099	0.00955	PYL	Abscisic acid receptor PYR/PYL family
c28880_g2	3.0377	1.37E-08	1.7311	0.030609	SAUR	SAUR family protein
c29262_g2	1.0321	0.03239	0.65653	0.001973	SNRK2	Serine/threonine-protein kinase SRK2
c30951_g1	−2.4569	5.55E-08	−1.1063	5.35E-07	SAUR	SAUR family protein
c31971_g1	−2.8478	3.08E-12	−1.144	7.84E-10	DELLA	DELLA protein
c32243_g1	1.5082	0.000256	0.63111	0.000377	EIN3	Ethylene-insensitive protein 3
c38393_g3	1.8563	5.93E-05	0.53353	0.006448	COI-1	Coronatine-insensitive protein 1
c39074_g1	2.236	0.00436	1.682	0.001339	GH3	Auxin responsive GH3 gene family
c42368_g2	−1.0309	0.02231	−0.54099	0.003132	PP2C	Protein phosphatase 2C
c43648_g4	3.5413	5.10E-06	0.61076	0.024029	NPR1	Regulatory protein NPR1

a *Log_2_FC: Log_2_(foldchange)*.

b *P adj: P adjusted*.

### qRT-PCR analysis

**Figure 5:**
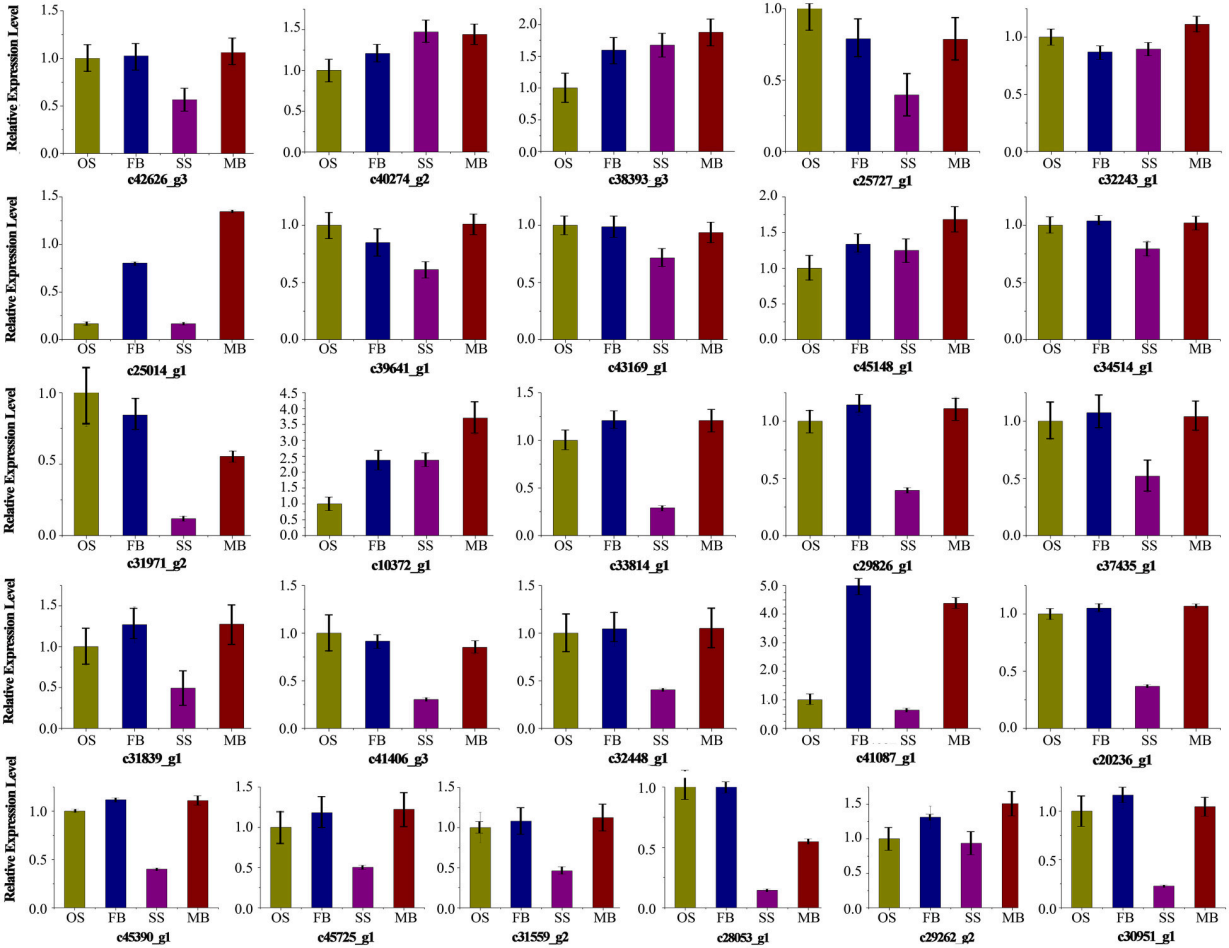
**Expression analysis of 26 genes in different samples**. Relative expression level in ovulate strobilus was set to 1.0. Error bars indicate the calculated maximum and minimum expression quantity of replicates. SS, staminate strobilus; OS, ovulate strobilus; MB, male bud; FB, female bud.

qRT-PCR was performed on 26 unigenes including 5 out of the 11 commonly differentially expressed unigenes involved in plant hormone signal and transduction (c29262_g2, c30951_g1, c31971_g1, c32243_g1, c38393_g3) to valid the expression profiling results obtained from the RNA-Seq data (Figure 5). The qRT-PCR and RNA-Seq results in the different organs were compared and the correlation between them was determined by calculating the correlation coefficient (*R*^2^). High correlation (*R*^2^ >0.9) was found between the RNA-Seq and qRT-PCR results, which indicated that the measured changes in gene expression detected by RNA-Seq reflected the actual transcriptome differences between the different libraries (Figure S6).

## Discussion

Sex determination is regulated at several points in the intricate network of involved genes in plants (Aryal and Ming, 2014). Moreover, genes known to be implicated in floral development (e.g., ABC model genes) do not have direct roles in sex determination (Hardenack et al., 1994; Ainsworth et al., 1995). It is well-appreciated that plant sex determination is mainly regulated by genes involved in phytohormone synthesis and transduction (Guo et al., 2010; Aryal and Ming, 2014; Zhang et al., 2014). As shown in Table 2, abscisic acid (ABA)-related genes encoding PP2C (protein phosphatase 2C, c42368_g2), and PYL (abscisic acid receptor 2, c27303_g1) that negatively regulated ABA transduction were down-regulated while one SNF1-related protein kinase subfamily 2 (*SNRK2*) gene that positively regulating ABA signaling was up-regulated in male organs. Antisense expression of *SNRK* gene in *Hordeum vulgare* interfered with pollen development, which caused male sterility (Zhang Y. et al., 2001). This may suggest that these proteins involved in ABA signaling in *G. biloba* is critical for reproduction development (Fujii and Zhu, 2009; Park et al., 2009). An auxin-reduced *SAUR* (*small auxin up RNA*) gene (c30951_g1) was down-regulated in male, two other *SAUR* genes (c19444_g1 and c28880_g2) and one *GH3* (Gretchen Hagen3) gene (c39074_g1) involved in auxin response showed higher expression level in male, which indicated that auxin related genes possessed diverse functions as in other plants (Kong et al., 2013). *Coronatine insensitive 1* (*COI1*), which involved in jasmonate (JA) perception and transduction in plants, showed higher expression level in male. Mutations of *COI1* in *Arabidopsis* displayed various degrees of male sterility (Ellis and Turner, 2002; Turner et al., 2002). The higher expression of *COI1* in male indicated that JA may play a crucial role in male reproductive development and sterility in *G. biloba*. Higher expression of *NPR1* (nonexpressor of PR gene) implicated in salicylic acid (SA) signaling in male may directly correlate with immunity to pathogens in *G. biloba* (Mou et al., 2003). Furthermore, interaction of these phytohormones may play important roles in *G. biloba* sex determination as in other developmental processes (Domagalska and Leyser, 2011; de Jong et al., 2014), further studies are needed to testify this hypothesis.

It is well-appreciated that transcription factors play important roles in regulating gene expression by temporarily and spatially regulating the transcription of their target genes (Hobert, 2008). Transcription factors such as a maize DELLA protein D8 and a melon zinc finger protein (CmW1P1) (Peng et al., 1999; Martin et al., 2009) have been shown to be associated with the sex determination process. Several transcription factors that belong to the C2C2-GATA, VOZ, and BZR1-BES1 families may play important roles in sex determination of cucumber (Guo et al., 2010). In the present study, a DELLA protein involved in gibberellin (GA) transduction (c31971_g1) and *ethylene-insensitive protein 3* (*EIN3*) (c32243_g1) showed simultaneously high expression level in male (Table 2). Ethylene plays a significant role in sex determination of plant species and can induce femaleness (Sisler, 1994). In cucumber, genes involved in ethylene biosynthesis and perception, as well as some ethylene-induced genes, have been found to be involved in sex determination (Boualem, 2009; Wu et al., 2010). Arabidopsis insensitive mutant, *ein3*, was defective in its ability to perceive or respond to ethylene (Solano et al., 1998). The higher expression of *EIN3* in male may inhibit the perceive and response to ethylene, which may result in male differentiation.

There is increasing evidence that DNA methylation is an important regulatory factor in plant sex determination. Furthermore, DNA methylation is hypothesized to be the first step toward the evolution of dioecy (Gorelick, 2003). Exploration of the methylation status in various species will help to understand the evolution of sex determination and sex chromosomes in plants (Aryal and Ming, 2014). In maize, sex determination was regulated by various small non-coding RNAs through DNA methylation (Parkinson et al., 2007; Hultquist and Dorweiler, 2008). Female sex suppression in *S. latifolia* males was depended on methylation of specific DNA sequences in the Y chromosome (Janoušek et al., 1996). In *Populus*, which has a ZW sex chromosome system, two genes related to DNA methylation were reported to be localized in the sex determining region of chromosome 19 (Song et al., 2013). At least 200 unigenes related to methyltransferase activity in GO enrichment analysis (e.g., GO0032259, GO0008168, GO0008649, see Table S5) showed higher expression level in female than those in male, which included genes that have been reported previously to be involved in plant sex determination, such as *MET1* and *COMT1* (Do et al., 2007; Schmidt et al., 2013). This is similar to the findings in *Populus* where more methylation sites were detected in female flowers compared with that in male flowers (Song et al., 2012). We suggested that the differentially expressed unigenes that encode proteins involved in DNA methylation may be related to sex determination in *G. biloba*. This result may help provide clues to the role of primary DNA methylation in sex determination in this species.

In plants, the formation of unisexual flowers can be classified mainly into two types. The first type includes *Asparagus officinalis* (Lazarte and Palser, 1979), cucumber (Bai et al., 2004), *Ceratonia siliqua* (Tucker, 1992), and *S. latifolia* ssp. *alba* (Ye et al., 1991) in which, at early developmental stages, male and female floral primordia are initially bisexual and contain primordial anthers and pistils. Sex determination appears to be the result of the selective arrest of pistillate primordial in male and of staminate primordial in female (Lazarte and Palser, 1979). Thus, sex determination genes do not become active at the moment of flower initiation when the different organ primordia appear, but at a crucial time during a later developmental stage (Caporali et al., 1994). The second type includes species such as *Spinacia oleracea* and *Mercurialis annua* (Sherry et al., 1993; Pannell, 1997), in which there are no traces of gynoecia in male flowers or of stamens in female flowers. Thus, sex determination occurs before inflorescence development, prior to or at evocation (Sherry et al., 1993). In MB, unigenes encoding pistil-specific extensin-like proteins were detected and expressed at a relatively high level (FPKM = 203.76). Similarly, in FB, unigenes encoding the pollen-specific protein SF21 and anther development proteins were detected (FPKM = 19.64 and 7.7 respectively). Furthermore, unigenes encoding apoptosis regulator proteins (e.g., metacaspase involved in regulation of apoptosis and apoptosis-related protein) and hydroxysteroid dehydrogenase (e.g., 3-beta hydroxysteroid dehydrogenase) were detected in both MB and FB (Table S6), which involved in triggering the organ death program and contributed to the selective abortion of the development of either the staminate or the pistillate primordia (DeLong et al., 1993). Based on these findings, we suggested that the formation of dioecism in *G. biloba* may resulted from the selective arrest of reproductive primordia and the unigenes implicated in the apoptosis pathway may play specific roles.

## Conclusions

In the present study, we sequenced and characterized the transcriptomes of *G. biloba* female and male buds and strobilus. The transcriptome resources that we obtained provided foundations for identifying functional genes related to sex determination in *G. biloba*. Based on these resources, we found that genes involved in plant hormone signal and those encoding DNA methyltransferase were differentially expressed between different sex types and dioecism in *G. biloba* was caused by the selective abortion of reproductive primordia. Concerted efforts with various model and non-model systems and studies over a pool of putative regulatory elements are necessary to further understand the complex mechanism of sex determination in plants.

## Author contributions

SX, YS designed the work, SD, XL, YS performed the experiment, analyzed the data and wrote the paper. HT, JL, LS collected the samples and revised it critically for important intellectual content.

### Conflict of interest statement

The authors declare that the research was conducted in the absence of any commercial or financial relationships that could be construed as a potential conflict of interest.

## Supplementary material

Figure S1 **Length distribution of the unigene sequences**.

Figure S2 **GO enrichment analysis of differentially expressed unigenes in MB vs. FB**. BP, Biological Process; CC, Cellular Component; MF, Molecular Function); MB, male bud; FB, female bud.

Figure S3 **GO enrichment analysis of differentially expressed unigenes in SS vs. OS**. BP, Biological Process; CC, Cellular Component; MF, Molecular Function; SS, staminate strobilus; OS, ovulate strobilus.

Figure S4 **KEGG enrichment analysis of differentially expressed unigenes in SS vs. OS**. SS, staminate strobilus; OS, ovulate strobilus.

Figure S5 **KEGG enrichment analysis of differentially expressed unigenes in MB vs. FB**. MB, male bud; FB, female bud.

Figure S6 **Correlation of the expression levels of 26 genes measured by qRT-PCR and RNA-seq**. SS: staminate strobilus; OS, ovulate strobilus; MB, male bud; FB, female bud.

Table S1 **Summary of RNA-Seq and *do novo* assembly results**.

Table S2 **Summary of the 26 genes in qRT-PCR analysis**.

Table S3 **Annotation results of the unigenes in public databases**.

Table S4 **Differentially expressed unigenes related to plant hormone signal and transduction**.

Table S5 **Differentially expressed unigenes related to DNA methyltransferase**.

Table S6 **Differentially expressed unigenes related to apoptosis regulator proteins and hydroxysteroid dehydrogenase**. MB, male bud; FB, female bud.

Data Sheet 1 **Unigenes sequences of the 11 commenly DEGs**.
