# Supplementary figures and images for: Transcriptome Profile Analysis from Different Sex Types of Ginkgo biloba L
Source: Front Plant Sci. 2016 Jun 16;7:871. doi: 10.3389/fpls.2016.00871 (PMC4910463; doi:10.3389/fpls.2016.00871)

Unigene Length Distribution

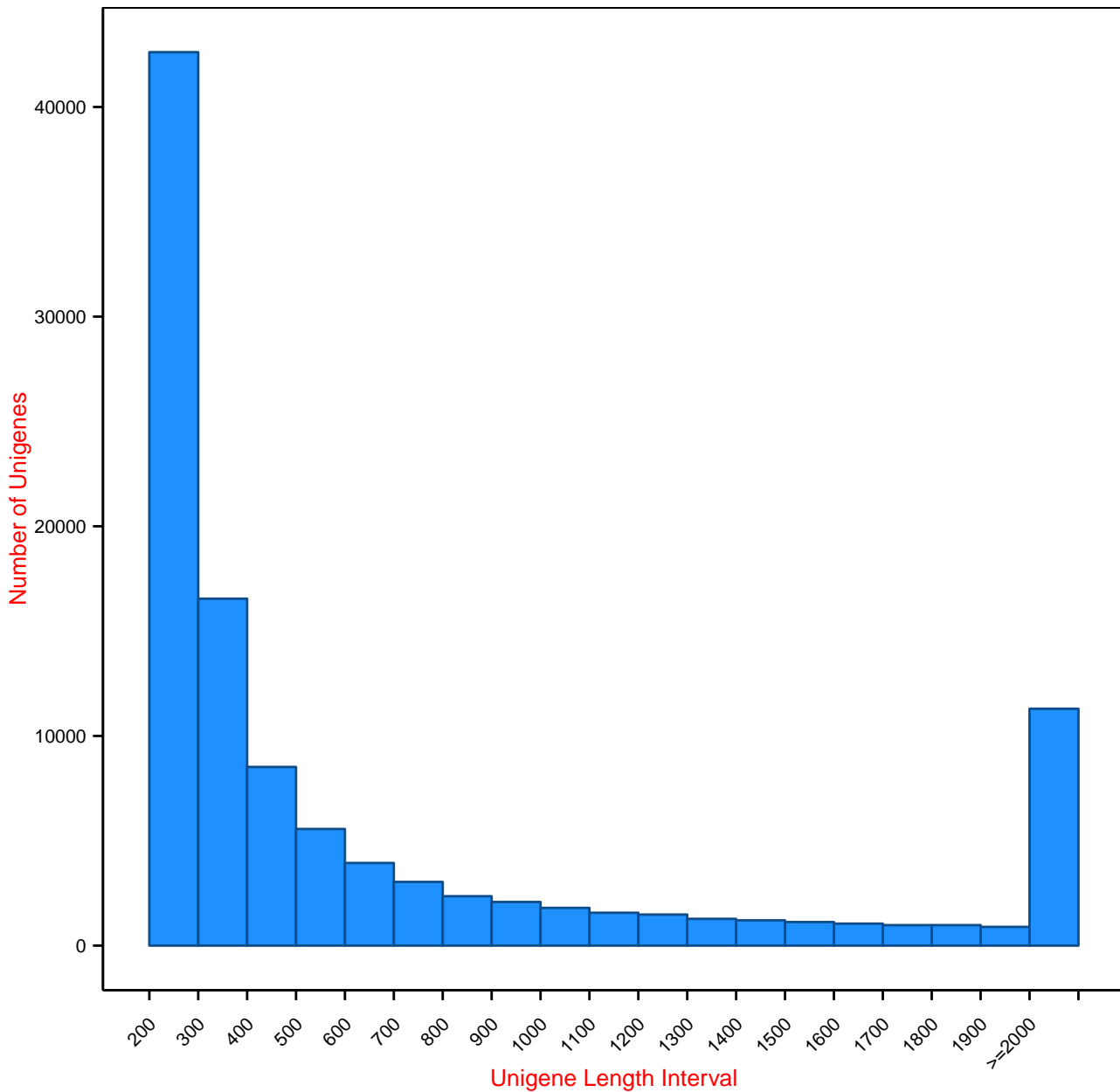

Supplement: Figure S1 — Length distribution of the unigene sequences. [file Image1.PDF]

Enriched GO Terms  
(MB vs. FB)

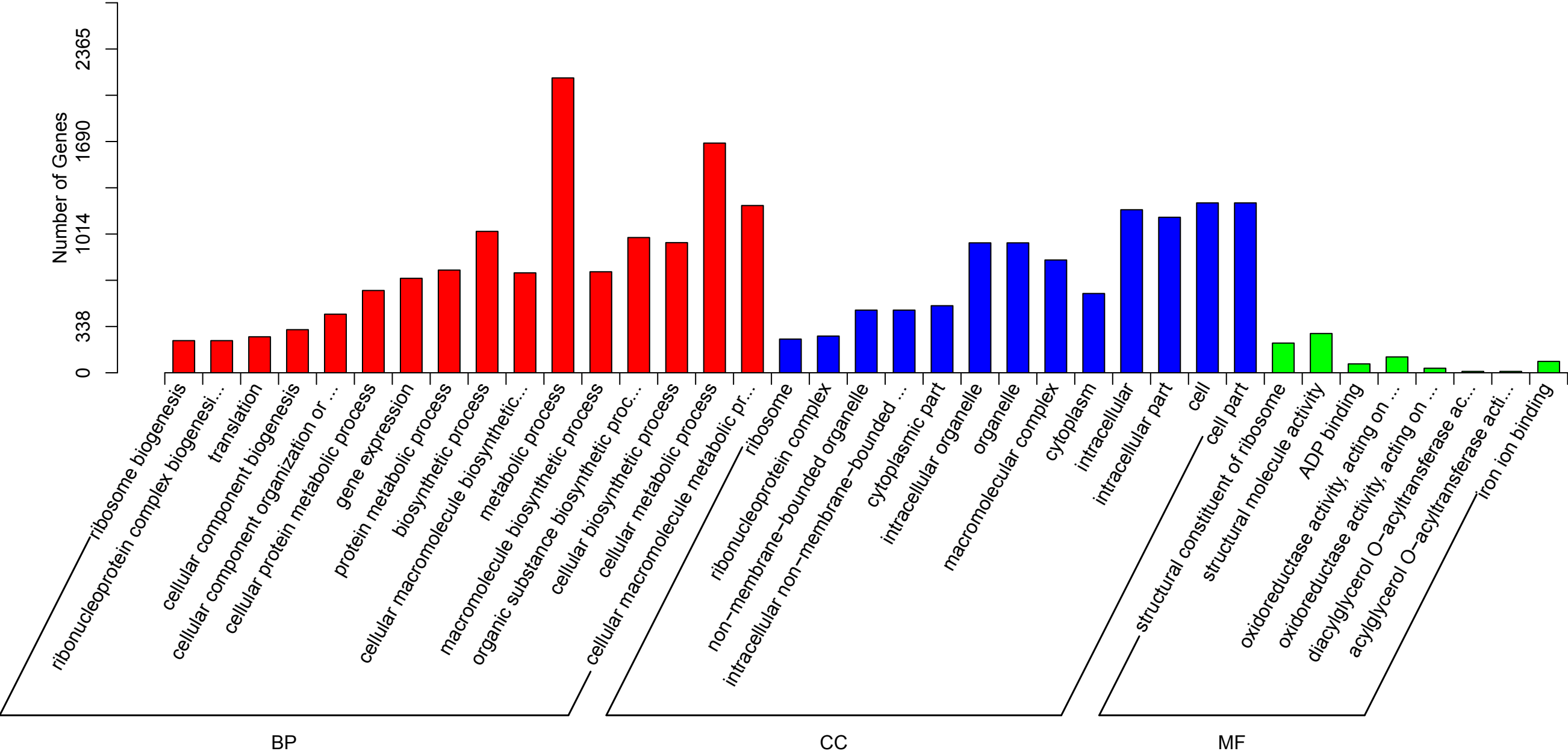

Supplement: Figure S2 — GO enrichment analysis of differentially expressed unigenes in MB vs. FB. BP, Biological Process; CC, Cellular Component; MF, Molecular Function); MB, male bud; FB, female bud. [file Image2.PDF]

# Enriched GO Terms (SS vs. OS)

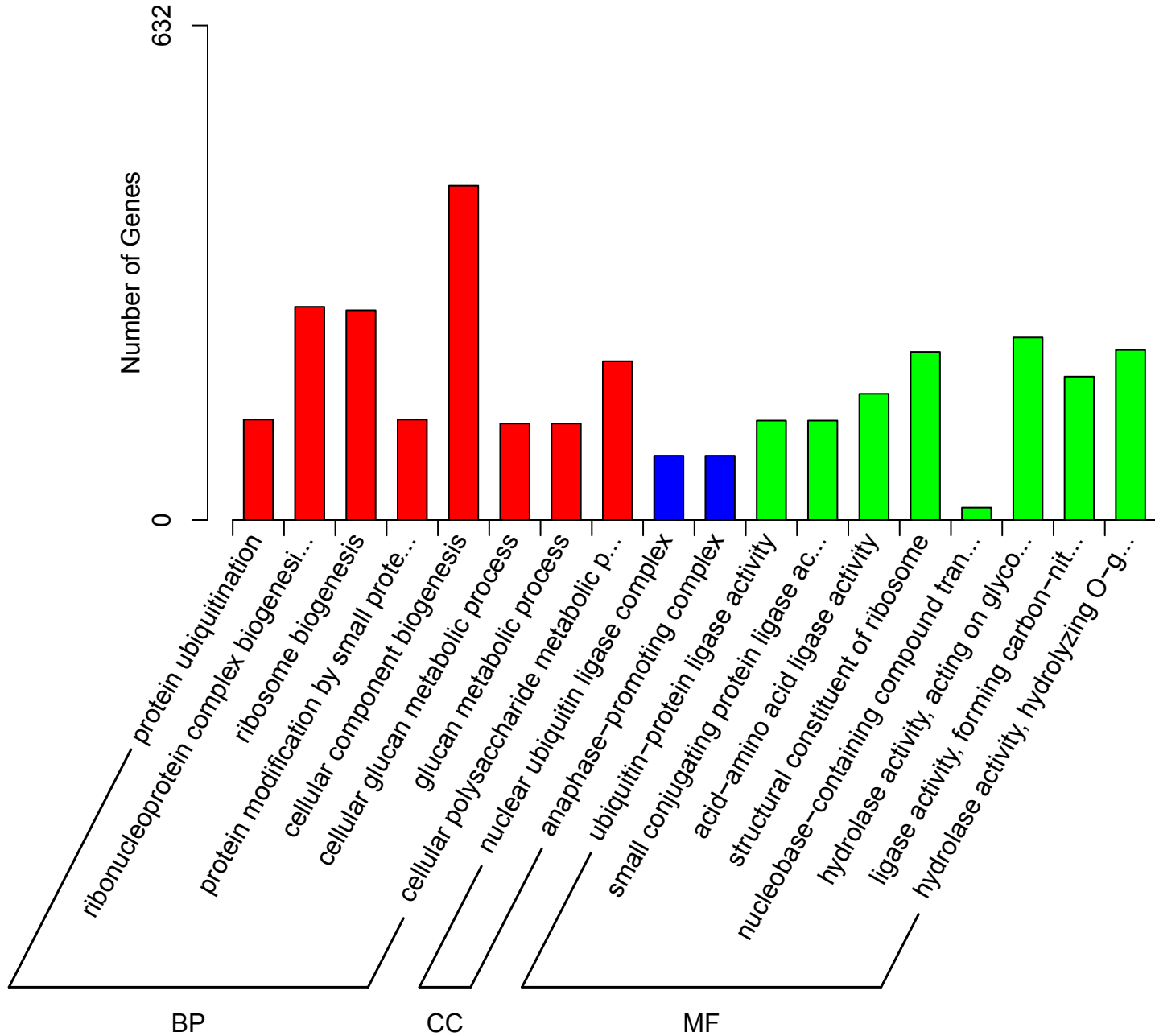

Supplement: Figure S3 — GO enrichment analysis of differentially expressed unigenes in SS vs. OS. BP, Biological Process; CC, Cellular Component; MF, Molecular Function; SS, staminate strobilus; OS, ovulate strobilus. [file Image3.PDF]

# Statistics of Pathway Enrichment

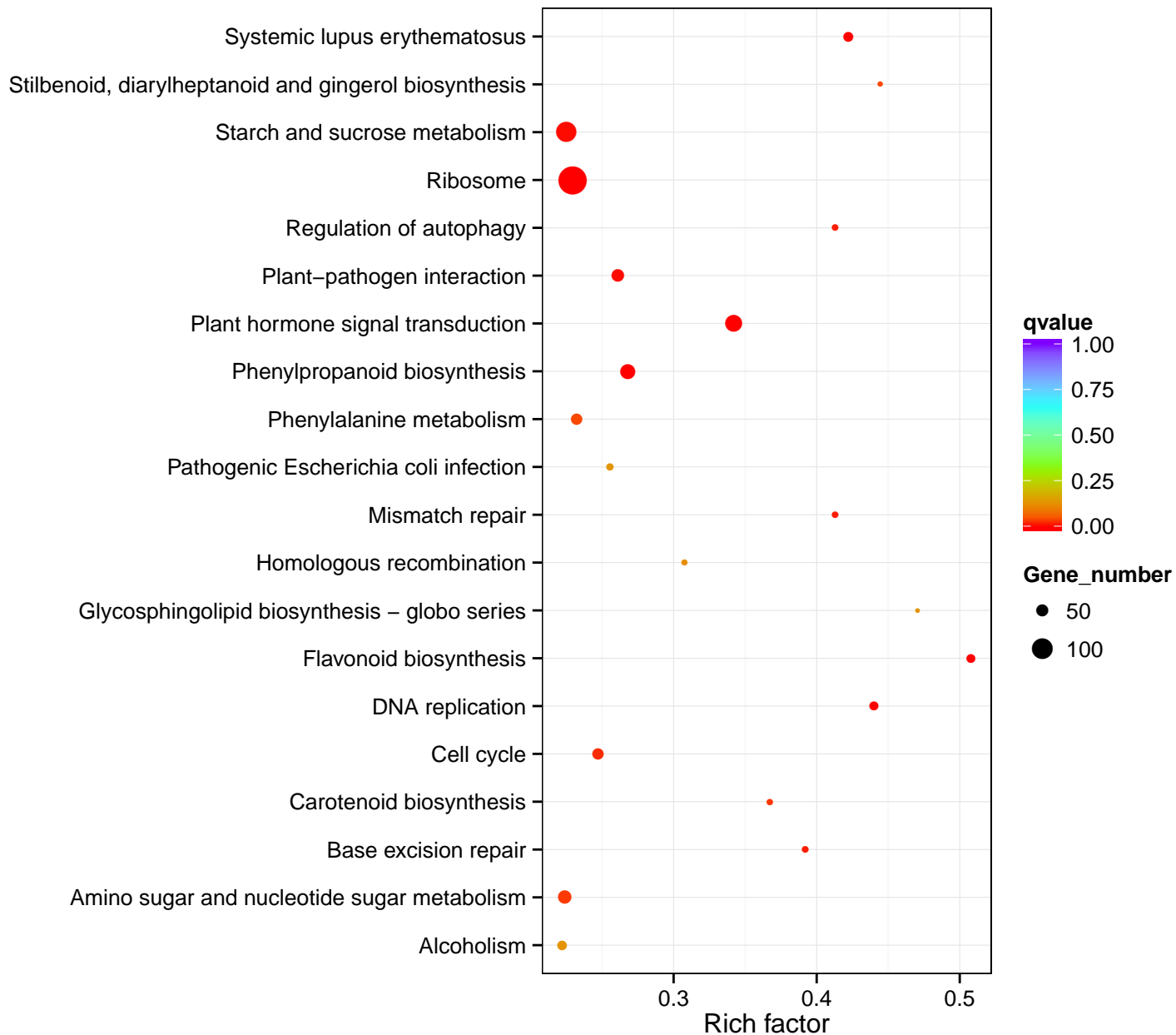

Supplement: Figure S4 — KEGG enrichment analysis of differentially expressed unigenes in SS vs. OS. SS, staminate strobilus; OS, ovulate strobilus. [file Image4.PDF]

# Statistics of Pathway Enrichment

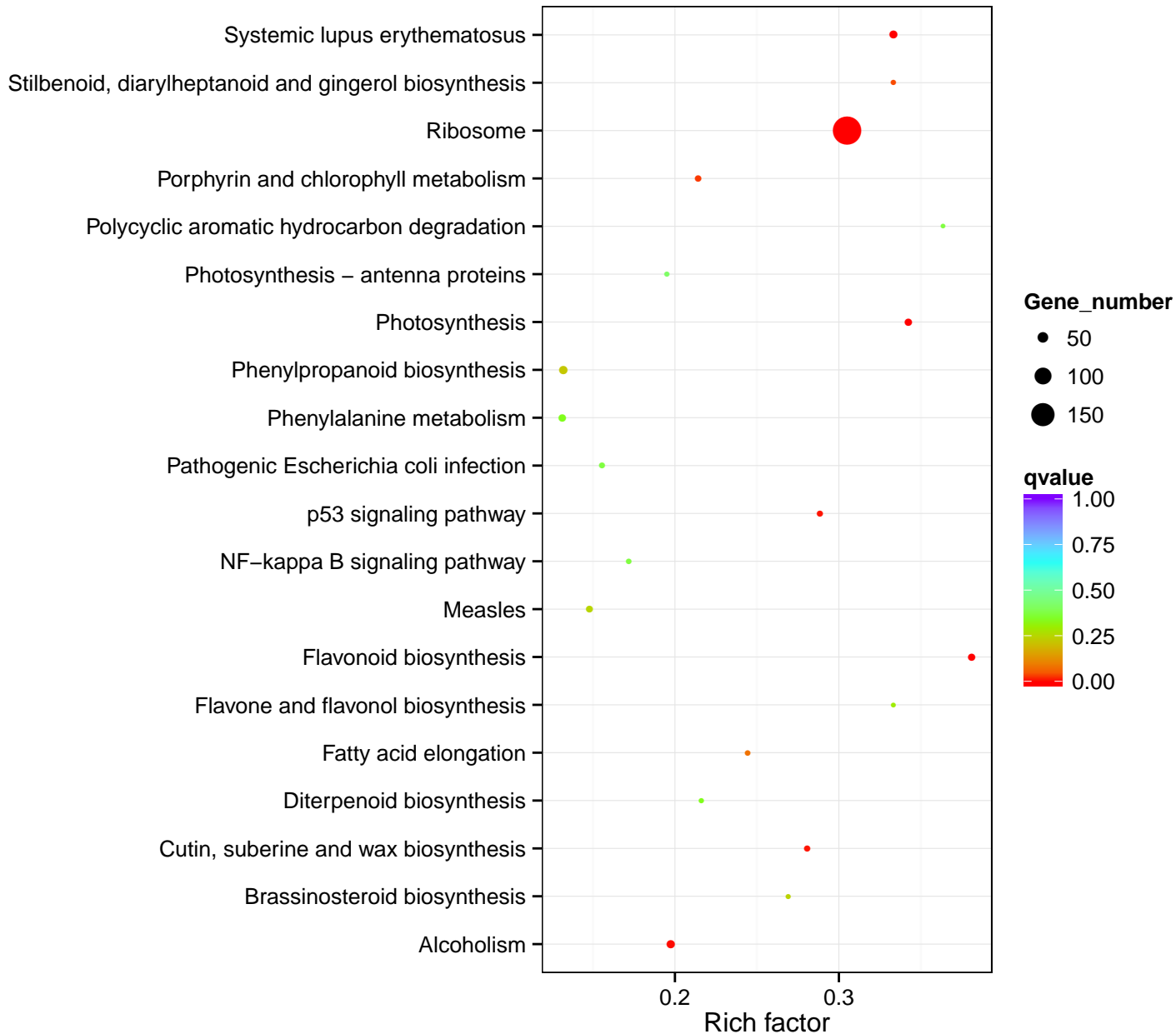

Supplement: Figure S5 — KEGG enrichment analysis of differentially expressed unigenes in MB vs. FB. MB, male bud; FB, female bud. [file Image5.PDF]

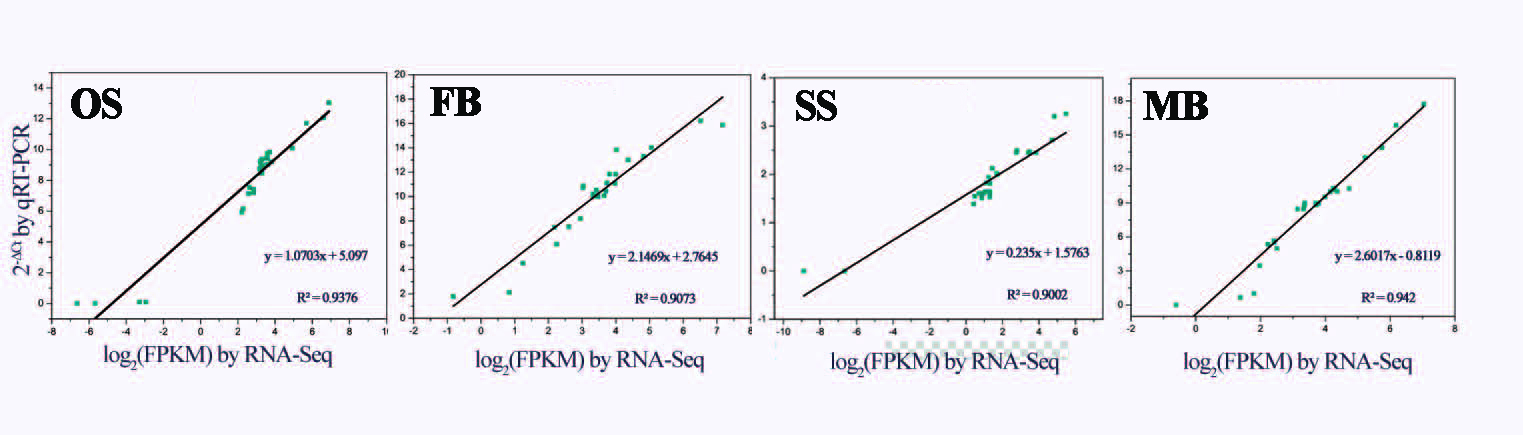

Supplement: Figure S6 — Correlation of the expression levels of 26 genes measured by qRT-PCR and RNA-seq. SS: staminate strobilus; OS, ovulate strobilus; MB, male bud; FB, female bud. [file Image6.JPEG]
