# Supplementary material for: Transcriptome Profile Analysis from Different Sex Types of Ginkgo biloba L
Source: Front Plant Sci. 2016 Jun 16;7:871. doi: 10.3389/fpls.2016.00871 (PMC4910463; doi:10.3389/fpls.2016.00871)
Supplement: Table S1 — Summary of RNA-Seq and do novo assembly results. [file Table1.DOCX]

S1 Table. Summary of RNA-Seq and *do novo* assembly results

| Sample | Raw Reads | Clean Reads | Clean Bases | Error (%) | Q20 (%) | Q30 (%) | GC Content （%） |
| --- | --- | --- | --- | --- | --- | --- | --- |
| MB | 122378298 | 119494172 | 14.94Gb | 0.03 | 96.02 | 92.24 | 44.13 |
| FB | 118278626 | 115958434 | 14.48Gb | 0.03 | 96.04 | 92.29 | 44.15 |
| SS | 124422188 | 121730470 | 15.22Gb | 0.04 | 95.33 | 91.09 | 44.32 |
| OS | 119364504 | 116504860 | 14.56Gb | 0.03 | 96.32 | 92.75 | 43.89 |
