# Supplementary material for: Transcriptome Profile Analysis from Different Sex Types of Ginkgo biloba L
Source: Front Plant Sci. 2016 Jun 16;7:871. doi: 10.3389/fpls.2016.00871 (PMC4910463; doi:10.3389/fpls.2016.00871)
Supplement: Table S2 — Summary of the 26 genes in qRT-PCR analysis. [file Table2.DOCX]

S2 Table Summary of the 26 genes in qRT-PCR analysis

| Gene ID | Functional annotation | qRT-PCR Primer (5'-3') | log_2_FPKM(OS) | 2^-ΔCt^ | log_2_FPKM(FB) | 2^-ΔCt^ | log_2_FPKM(SS) | 2^-ΔCt^ | log_2_FPKM(MB) | 2^-ΔCt^ |
| --- | --- | --- | --- | --- | --- | --- | --- | --- | --- | --- |
| c42626_g3 | auxin response factor | F:GCCATCGAAGACAACTAAA | 4.93 | 10.08 | 5.05 | 14.03 | 2.79 | 2.49 | 5.23 | 13.02 |
|  |  | R:CAAGCATCATATCACCCTC |  |  |  |  |  |  |  |  |
| c40274_g2 | PHYB activation tagged  suppressor 1 | F:AAGAAATGAAGCTGGGAAAC | 3.29 | 9.38 | 3.97 | 11.08 | 4.83 | 3.20 | 4.73 | 10.28 |
|  |  | R:GGAATGAATGCCAAAGGA |  |  |  |  |  |  |  |  |
| c38393_g3 | coronatine-insensitive  protein 1 | F:GATGAAGGTGGTGAATGG | 2.29 | 6.16 | 3.65 | 10.06 | 3.83 | 2.45 | 4.29 | 10.16 |
|  |  | R:ATCAACTTTGCAGGGAAT |  |  |  |  |  |  |  |  |
| c25727_g1 | DNA (cytosine-5)-  methyltransferase | F:CCTTATGCGAAAGACTGAG | 2.84 | 7.20 | 2.23 | 6.09 | 1.12 | 1.82 | 2.22 | 5.36 |
|  |  | R:TCTGGACCTACCCAAACTA |  |  |  |  |  |  |  |  |
| c32243_g1 | ethylene-insensitive protein 3 | F:CCTTATGCGAAAGACTGAG | 3.81 | 9.21 | 3.32 | 10.14 | 3.41 | 2.45 | 4.24 | 10.29 |
|  |  | R:TCTGGACCTACCCAAACTA |  |  |  |  |  |  |  |  |
| c25014_g1 | F-box protein GID2 | F:ATCTGGATGCCAAGTCGC | -6.64 | 0.01 | -0.82 | 1.73 | -6.64 | 0.01 | 1.37 | 0.65 |
|  |  | R:AGTGCCATCACAACCCTC |  |  |  |  |  |  |  |  |
| c39642_g1 | jasmonate ZIM domain-  containing protein | F:CAATGGGACGGTGAATGT | 5.68 | 11.71 | 4.82 | 13.30 | 3.48 | 2.47 | 5.74 | 13.89 |
|  |  | R:AGCGGGAAATGTTGAGAT |  |  |  |  |  |  |  |  |
| c43169_g1 | DNA (cytosine-5)-  methyltransferase 1 | F:AGCAGAGGATACAGCCATTA | 6.60 | 12.06 | 6.51 | 16.22 | 4.71 | 2.71 | 6.17 | 15.85 |
|  |  | R:AGAGTAGCCCGTGTCATA |  |  |  |  |  |  |  |  |
| c45148_g1 | transcription factor MYC2 | F:CGAATCCCGCTGGTATCCT | 2.21 | 5.93 | 2.94 | 8.18 | 2.76 | 2.45 | 3.71 | 8.83 |
|  |  | R:CCTCGTTTCCGTGGCTTTT |  |  |  |  |  |  |  |  |
| c34514_g1 | protein arginine N-  methyltransferase 1 | F:GGACCTAAATCTCGTGCTA | 6.90 | 13.03 | 7.16 | 15.88 | 5.47 | 3.25 | 7.04 | 17.71 |
|  |  | R:GGTTCCAACAAGTGCCTC |  |  |  |  |  |  |  |  |
| c31971_g2 | DELLA | F:ACAGTGCTCCCGTCTTCT | 3.58 | 9.74 | 3.01 | 10.72 | 0.42 | 1.39 | 1.98 | 3.47 |
|  |  | R:TTCGTGCCTTTCTACCCT |  |  |  |  |  |  |  |  |
| c10372_g1 | SAUR family protein | F:GGAATACGGGATAGACCAG | -2.94 | 0.10 | 1.23 | 4.53 | 1.23 | 1.94 | 2.41 | 5.67 |
|  |  | R:GACCAACCCTAGCACATAC |  |  |  |  |  |  |  |  |
| c33814_g1 | transport inhibitor response 1 | F:GCTGACTGAACTAGGCACT | 3.61 | 9.03 | 4.36 | 13.01 | 1.05 | 1.64 | 4.36 | 10.02 |
|  |  | R:GAACAGGCAGAATAAATGG |  |  |  |  |  |  |  |  |
| c29826_g1 | Apoptosis inhibitor IAP1 and related BIR domain proteins | F:CGGGACTTAACCCAACACCTT | 3.33 | 8.98 | 3.81 | 11.84 | 1.31 | 1.81 | 3.70 | 9.01 |
|  |  | R:GTCGCAAGACTGAAACTCA |  |  |  |  |  |  |  |  |
| c37435_g1 | Apoptosis-inducing factor homolog A | F:CTAACCTTGCGTCAGGACC | 3.23 | 9.05 | 3.47 | 10.01 | 1.67 | 2.02 | 3.36 | 8.91 |
|  |  | R:ACGATCTGGGCACTGTCTC |  |  |  |  |  |  |  |  |
| c31839_g1 | 3-beta hydroxysteroid dehydrogenase | F:GGAGGTGGTGGAGGAAGTG | 2.62 | 7.54 | 3.33 | 10.01 | 1.29 | 1.64 | 3.34 | 8.75 |
|  |  | R:CCGCCTGATGAGGATGACGA |  |  |  |  |  |  |  |  |
| c41406_g3 | ribulose-bisphosphate carboxylase small chain | F:GCGTCTATGCTTTCCTCTTC | 2.84 | 7.41 | 2.60 | 7.51 | 0.86 | 1.50 | 2.42 | 5.49 |
|  |  | R:AAGGCAACTTCCACATTGTCC |  |  |  |  |  |  |  |  |
| c32448_g1 | 3-beta hydroxysteroid dehydrogenase | F:TGGCAGTTACTGACTTTGGA | 3.20 | 9.23 | 3.34 | 10.14 | 1.29 | 1.63 | 3.36 | 9.01 |
|  |  | R:TGATACGGTCTGGTATTGGA |  |  |  |  |  |  |  |  |
| c41087_g1 | Apoptosis inhibitor IAP1 and related BIR domain proteins | F:TATCAAAACTCAGAAGGTCC | -5.67 | 0.01 | 0.83 | 2.12 | -8.89 | 0.01 | -0.60 | 0.01 |
|  |  | R:GCAAATGTACAGTAATATG |  |  |  |  |  |  |  |  |
| c20236_g1 | CodY GAF-like domain | F:GGCGAGATTACCTTTGATT | 3.55 | 9.43 | 3.73 | 11.12 | 1.30 | 1.62 | 3.79 | 8.94 |
|  |  | R:GCGTCTTGGCTAGGATTT |  |  |  |  |  |  |  |  |
| c45390_g1 | Apoptosis inhibitor IAP1 and related BIR domain proteins | F:ATCTAATCCCATTCGCTCCC | 3.59 | 9.54 | 4.02 | 13.84 | 1.43 | 2.12 | 3.98 | 9.53 |
|  |  | R:TTCAAGTCCGCCGTCAAA |  |  |  |  |  |  |  |  |
| c45725_g1 | apoptosis inhibitor 5-like | F:TTTTGTGCTTGTGCATAAACCAT | 2.56 | 7.13 | 3.03 | 10.87 | 1.29 | 1.53 | 3.14 | 8.45 |
|  |  | R:CTATGAAGGGAAACCAACCAGAC |  |  |  |  |  |  |  |  |
| c31559_g2 | Metacaspase involved in regulation of apoptosis | F:GAACATTTCGTATCTCGTAG | 3.70 | 9.84 | 3.98 | 11.84 | 1.70 | 1.99 | 4.14 | 9.98 |
|  |  | R:AGAGGATTCATAAGGGTAA |  |  |  |  |  |  |  |  |
| c28053_g1 | regulation of apoptotic process | F:CGATAGCGAAGTAGTACCGTGAG | 3.28 | 8.46 | 3.42 | 10.53 | 0.47 | 1.54 | 1.79 | 1.02 |
|  |  | R:CACCGAAACAGTGCTTTACCC |  |  |  |  |  |  |  |  |
| \| c29262_g2 \| \| --- \| | serine/threonine-protein kinase SRK2 | F:GGAAGTTGTCGGTGCTCGTCT | -3.29 | 0.10 | 2.18 | 7.45 | 0.95 | 1.61 | 2.50 | 4.98 |
|  |  | R:AAGGGTTGTCGCCAAGGTTAT |  |  |  |  |  |  |  |  |
| c30951_g1 | SAUR family protein | F:CTTACTGAAAGAGTTGGAGGTT | 3.17 | 8.74 | 3.70 | 10.45 | 0.71 | 1.60 | 3.32 | 8.45 |
|  |  | R:CCTTCCCAGTTTGCTTCT |  |  |  |  |  |  |  |  |
